# Immune Checkpoint Inhibitor-Related Hepatitis Versus Classical Autoimmune Hepatitis: A Retrospective Multidomain Phenotype Comparison

**DOI:** 10.3390/jcm15197572

**Published:** 2026-09-29

**Authors:** Mehmet Kursad Keskin, Alper Coskun, Nesrin Ugras, Erdem Cubukcu, Selim Giray Nak

**Affiliations:** 1 Department of Gastroenterology, Faculty of Medicine, Bursa Uludağ University, Bursa 16059, Türkiye; 2 Department of Medical Oncology, Faculty of Medicine, Bursa Uludağ University, Bursa 16059, Türkiye; 3 Department of Medical Pathology, Faculty of Medicine, Bursa Uludağ University, Bursa 16059, Türkiye

**Keywords:** immune checkpoint inhibitors, immune-related hepatitis, autoimmune hepatitis, drug-induced liver injury, phenotype, liver biopsy, RUCAM

## Abstract

**Background/Objectives**: Immune checkpoint inhibitor-related hepatitis (ICI-H) can resemble classical autoimmune hepatitis (AIH), but comparative multidomain data remain limited. We compared their clinical, biochemical, and serological profiles and descriptively summarized histopathological findings. **Methods**: This single-centre retrospective cohort screened 100 records from January 2018 to October 2023; 79 patients met predefined criteria (40 ICI-H; 39 AIH). ICI-H required an updated RUCAM score of ≥6, whereas AIH required a simplified AIH score of ≥6 and compatible histology. Continuous and categorical variables were assessed using Mann–Whitney U and Fisher’s exact tests with Benjamini–Hochberg correction where applicable; histopathological findings were summarized descriptively because biopsy ascertainment was markedly unequal between groups. **Results**: Compared with AIH, ICI-H occurred more often in men (65.0% vs. 20.5%) and showed higher AST, ALT, ALP, GGT, INR, CRP, and neutrophil-to-lymphocyte ratio (all q < 0.001), but lower ANA positivity (12.5% vs. 74.4%; q < 0.001). Histopathological observations were limited to the biopsy-assessed subgroup (10 ICI-H; 39 AIH) and are reported descriptively because biopsy ascertainment was markedly unequal and non-random. **Conclusions**: ICI-H and classical AIH showed different observed clinical, biochemical, and serological profiles; however, these differences should not be interpreted as independent diagnostic discriminators because disease-specific criteria contributed to group assignment. Diagnosis after ICI exposure should integrate exposure timing, exclusion of competing causes, structured causality assessment, serology, and selective histology. Outcome comparisons require standardized longitudinal follow-up.

## 1. Introduction

Immune checkpoint inhibitors (ICIs) strengthen antitumour immunity by blocking inhibitory pathways such as cytotoxic T-lymphocyte-associated protein 4, programmed cell death protein 1, and programmed death-ligand 1. Their clinical benefit is accompanied by immune-related adverse events that can involve almost any organ. Hepatic toxicity ranges from asymptomatic biochemical abnormalities to hepatitic, cholangitic, or mixed injury and, rarely, acute liver failure. Incidence and severity vary across drug classes and are highest with combination immunotherapy [1,2,3,4,5].

ICI-related hepatitis (ICI-H), also termed checkpoint inhibitor-induced liver injury, has no pathognomonic clinical or laboratory marker. The diagnosis depends on the temporal relationship with ICI exposure and the exclusion of other causes, including tumour progression or hepatic infiltration, biliary obstruction, viral infection, sepsis, ischaemia, alcohol-related disease, and injury from concomitant drugs. Toxicity grading is useful for immediate management, but the degree of aminotransferase elevation does not necessarily identify the mechanism or predict the clinical course [3,4]. ICI-H is therefore better considered a heterogeneous immune-mediated drug-induced liver injury than a direct counterpart of classical autoimmune hepatitis (AIH).

Classical AIH is a chronic immune-mediated liver disease that predominantly affects women. Its diagnosis integrates aminotransferase activity, serum immunoglobulin G (IgG), conventional autoantibodies, compatible histology, and exclusion of competing disorders. Antinuclear antibody (ANA), anti-smooth muscle antibody (ASMA), anti-liver kidney microsomal type 1 antibody, and anti-soluble liver antigen/liver-pancreas antibody contribute to classification but are not diagnostic alone. The revised International Autoimmune Hepatitis Group (IAIHG) system and the simplified score formalise this assessment [6,7]. Even after treatment, relapse and progression to cirrhosis, decompensation, or liver transplantation remain important clinical concerns [6,8].

ICI-H and classical AIH share aminotransferase elevation, immune-mediated hepatocyte injury, and frequent corticosteroid use, but their serological profiles differ. Conventional autoantibodies are less common and less disease-specific in ICI-H, and IgG is often normal or only moderately increased. Autoantibodies detected before or during ICI treatment have not reliably predicted hepatic toxicity [9]. By contrast, ANA or ASMA positivity, hypergammaglobulinaemia, and higher AIH diagnostic scores more often support classical AIH [9,10,11].

Histology also suggests that the two conditions are not equivalent. Classical AIH commonly shows portal and interface hepatitis with plasma-cell-rich inflammation, hepatocyte rosettes, and, in some cases, emperipolesis. ICI-H has a broader spectrum that includes lobular or centrilobular hepatitis, spotty or confluent necrosis, granulomatous inflammation, endothelialitis, eosinophils, and bile-duct injury; prominent plasma cells may be absent [10,11,12,13,14]. Reports of CD8-positive cytotoxic T-cell predominance, including a circulating exhausted effector-memory population, further distinguish ICI-associated injury from classical AIH [15].

The treatment course also differs. Classical AIH usually requires induction of remission followed by maintenance immunosuppression, and relapse after withdrawal is common [6,8]. Management of ICI-H begins with interruption of immunotherapy and is guided by clinical severity. Corticosteroids are frequently used, while inadequate responses may prompt a second immunosuppressant, most often mycophenolate mofetil [2,3,13,16]. Longer-term hepatic outcomes and the effect of immunosuppression on cancer control are less well defined.

Comparative cohorts that evaluate ICI-H and classical AIH across clinical, biochemical, serological, and histological domains remain limited [10,11,12]. Previous comparative studies have predominantly focused on histopathological characteristics, included additional comparator groups—such as conventional drug-induced liver injury or other autoimmune liver diseases—and/or evaluated relatively small numbers of ICI-associated cases. The present study was therefore designed to complement the existing literature through a direct multidomain comparison of ICI-H and classical AIH, incorporating clinical, biochemical, serological, inflammatory, and histopathological characteristics together with structured updated RUCAM-based causality assessment for ICI-H. Furthermore, comparative data from Türkiye and the surrounding region remain limited, and this cohort broadens the geographic representation of the available evidence. Our primary objective was to characterize phenotype-level differences between ICI-H and classical AIH, while treatment characteristics and clinically recorded outcomes were considered secondary descriptive domains because longitudinal follow-up was not uniformly available.

## 2. Materials and Methods

### 2.1. Study Design and Setting

This single-centre retrospective observational cohort study was conducted at the Department of Gastroenterology, Bursa Uludağ University Faculty of Medicine, Bursa, Türkiye. Records from patients receiving routine care between January 2018 and October 2023 were reviewed.

The study was reported in accordance with the Strengthening the Reporting of Observational Studies in Epidemiology (STROBE) statement [17].

The record-review window defined eligibility for the source population, and all included index hepatitis episodes occurred between January 2018 and October 2023. Baseline variables were reconstructed from the original diagnostic episode. All data arose from routine care; there was no research-specific visit, intervention, or specimen collection.

**Figure 1:**
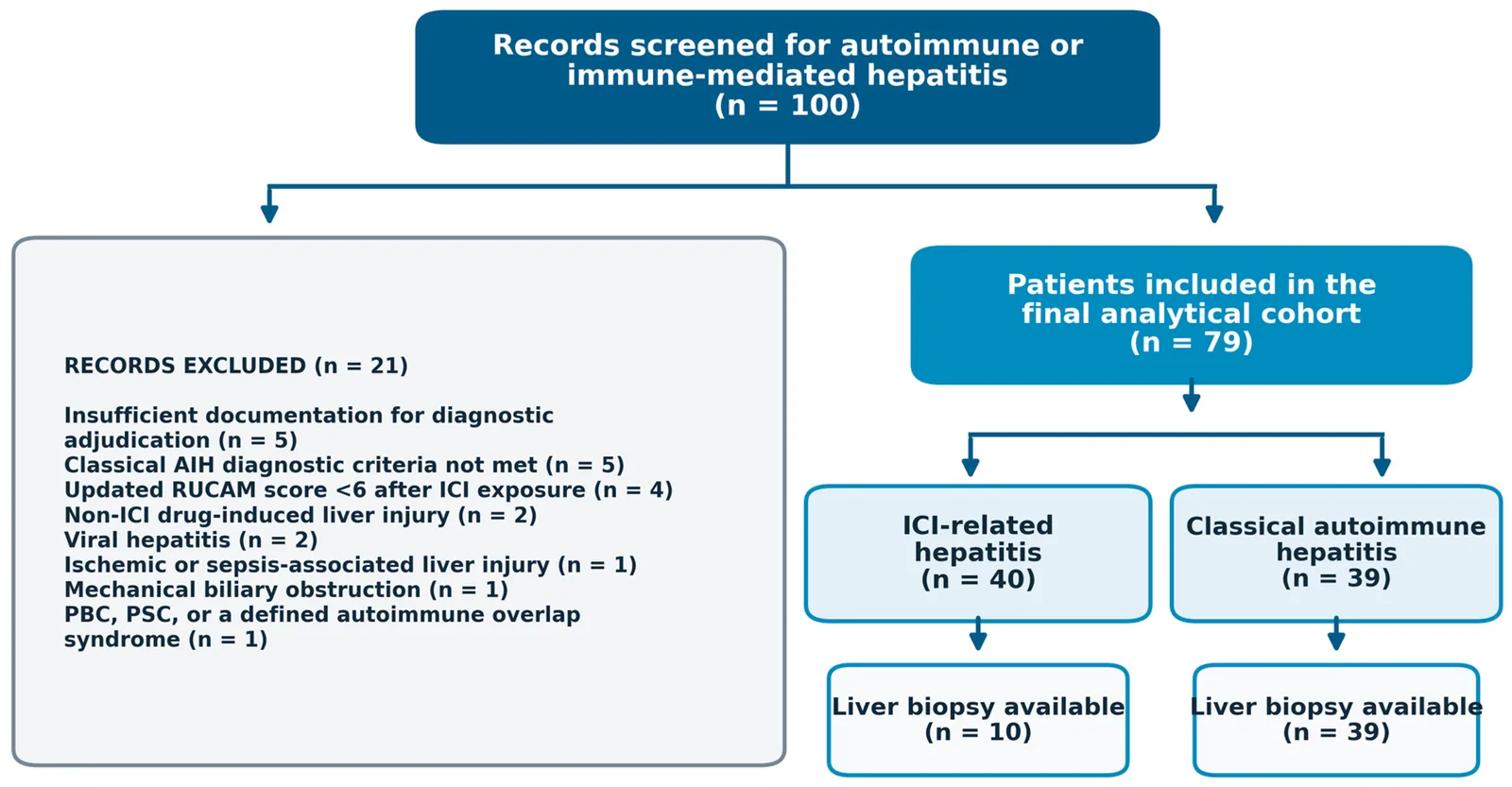
Study flow. Of 100 screened records, 21 were excluded according to mutually exclusive primary reasons. The final analytical cohort comprised 79 patients: 40 with ICI-related hepatitis and 39 with classical autoimmune hepatitis. Liver biopsy data were available for 10 and 39 patients, respectively.

Of the 100 records screened, 21 were excluded. The reasons for exclusion were insufficient documentation for diagnostic adjudication (*n* = 5), failure to meet the predefined diagnostic criteria for classical AIH (*n* = 5), an updated RUCAM score of <6 following ICI exposure (*n* = 4), non-ICI drug-induced liver injury (*n* = 2), viral hepatitis (*n* = 2), ischemic or sepsis-associated liver injury (*n* = 1), mechanical biliary obstruction (*n* = 1), and cholestatic autoimmune liver disease or a defined overlap syndrome (*n* = 1). Consequently, 79 patients were included in the final analysis, comprising 40 patients with ICI-H and 39 with classical AIH (Figure 1). The four ICI-exposed patients excluded because of an updated RUCAM score of <6 were additionally reviewed at the individual-patient level, and their ICI exposure chronology and biochemical characteristics are provided in Supplementary Table S1.

### 2.2. Eligibility Criteria and Diagnostic Classification

Patients were eligible if they were aged ≥18 years, had sufficient medical-record information to establish the cause of liver injury, and fulfilled the predefined diagnostic criteria for either ICI-H or classical AIH.

#### 2.2.1. Immune Checkpoint Inhibitor-Related Hepatitis

The ICI-H group included patients who had the following conditions:

1. Having received at least one dose of an immune checkpoint inhibitor.
2. Having developed new-onset biochemical liver injury after ICI exposure.
3. Having undergone an appropriate evaluation for competing causes of liver injury.
4. Having an updated Roussel Uclaf Causality Assessment Method (RUCAM) score of ≥6, indicating at least probable causality [18,19,20].

The RUCAM ≥ 6 threshold was retained as the predefined eligibility criterion for the primary ICI-H cohort and was not modified after review of the excluded cases.

The differential diagnostic evaluation included viral hepatitis, other hepatotoxic medications or herbal products, alcohol-related liver injury, biliary obstruction, hepatic tumour progression or infiltration, liver metastases, vascular or ischaemic injury, sepsis, and pre-existing autoimmune or chronic liver disease. Imaging findings and the clinical course were considered when excluding biliary obstruction, vascular disease, or progression of hepatic malignancy. Patients with a documented diagnosis of classical AIH preceding ICI exposure were not classified as having ICI-H.

Index-episode transaminase severity in the ICI-H group was graded according to the National Cancer Institute Common Terminology Criteria for Adverse Events (CTCAE), version 5.0 [21], using the laboratory upper limits of normal for AST (35 U/L) and ALT (45 U/L). Because baseline aminotransferase levels were within the normal range in all ICI-H patients, grading was based on multiples of the upper limit of normal. Simplified and revised IAIHG scores were calculated to describe AIH-related diagnostic features but were not used to reclassify ICI-H. To reduce incorporation and availability bias, score summaries were restricted to patients with an available biopsy-based assessment and were not subjected to between-group hypothesis testing.

#### 2.2.2. Classical Autoimmune Hepatitis

Classical AIH was classified by integrating presentation, aminotransferase activity, serum IgG, autoantibody findings, exclusion of competing liver diseases, and compatible histology, using IAIHG criteria and current practice guidance [6,7,22,23,24].

Patients in the classical AIH group were required to have a simplified AIH score of ≥6, corresponding to probable or definite AIH, together with compatible clinicopathological assessment. The revised original IAIHG score was additionally calculated from pretreatment variables when available. Liver biopsy findings were available for all classical AIH patients. Because the simplified score contributed to cohort eligibility, neither diagnostic score was treated as an inferential comparative outcome.

Patients were excluded in the presence of an inadequately investigated liver enzyme elevation, active viral hepatitis explaining the liver injury, biliary obstruction, ischaemic or congestive hepatitis, a more probable non-ICI drug-induced liver injury, an autoimmune cholestatic overlap syndrome without a predominant AIH component, duplicate records, or insufficient core data for diagnostic classification. Because group assignment relied on disease-specific diagnostic criteria that incorporate some of the same clinical, biochemical, serological, and histological domains subsequently summarized, between-group differences were interpreted as descriptive cohort characteristics rather than as independent diagnostic discriminators.

### 2.3. Data Collection and Definition of the Index Episode

Data were abstracted retrospectively from electronic medical records, laboratory databases, oncology treatment files, discharge summaries, radiology reports, and routine liver pathology reports.

The index episode was defined as the first documented episode at which ICI-H or classical AIH was diagnosed. Laboratory values were taken from the measurement closest to the index diagnosis and before corticosteroid or other immunosuppressive treatment when pretreatment timing could be verified; otherwise, the value was treated as unavailable for pretreatment analyses.

The following variables were abstracted:

- Demographic and clinical characteristics: age, sex, body mass index, diabetes mellitus, hypertension, cirrhosis at index diagnosis, acute or chronic presentation, and duration of follow-up.
- Oncology and ICI-related characteristics: primary malignancy, cancer stage, liver metastasis, ICI class and agent, number of treatment cycles, interval from ICI initiation to hepatitis, concurrent immune-related adverse events, ICI discontinuation, rechallenge, and recurrence following rechallenge.
- Biochemical parameters: aspartate aminotransferase, alanine aminotransferase, alkaline phosphatase, gamma-glutamyl transferase, total and direct bilirubin, international normalised ratio, albumin, platelet count, white blood cell count, absolute neutrophil and lymphocyte counts, C-reactive protein, ferritin, and immunoglobulin G.
- Serological parameters: antinuclear antibody, anti-smooth muscle antibody, antimitochondrial antibody, anti-liver kidney microsomal antibody, anti-soluble liver antigen/liver–pancreas antibody, and perinuclear antineutrophil cytoplasmic antibody.
- Infectious markers: hepatitis A, B, C, and E markers and, where clinically indicated, cytomegalovirus and Epstein–Barr virus testing.
- Treatment variables: corticosteroid administration, corticosteroid type and initial dose, intravenous corticosteroid use, time to corticosteroid initiation, azathioprine, mycophenolate mofetil, tacrolimus, ursodeoxycholic acid, and treatment duration.
- Clinical outcomes: early biochemical response, complete biochemical response, steroid-refractory disease, requirement for second-line immunosuppression, relapse, acute liver failure, hospitalisation, intensive care unit admission, liver transplantation, all-cause death, liver-related death, and cancer progression.

A serological test documented as “not performed” was retained as missing and was not classified as negative.

### 2.4. Data Standardisation and Quality Control

All records were assigned anonymised study identifiers before analysis. Dates, categorical codes, laboratory units, and missing-value definitions were standardized using a predefined data dictionary. Laboratory values were converted to common units before calculation of diagnostic descriptors or derived inflammatory indices.

Missing values were not replaced by zero and were not interpreted as absence of the relevant clinical finding. No statistical imputation was undertaken. Analyses were conducted using available cases, and the denominator for each variable was reported where missing data were present.

Numerical values outside prespecified clinically credible ranges were not winsorised or replaced by estimated values. Values that could not be verified or interpreted reliably were treated as missing. Derived indices were calculated only when all required components were available.

### 2.5. Histopathological Evaluation

Liver biopsy was performed as part of routine clinical care according to the treating physician’s assessment. Biopsy was not required for inclusion in the ICI-H group when the temporal association, exclusion of competing causes, and causality assessment supported the diagnosis.

Histopathological information was available for 10 of the 40 patients with ICI-H and all 39 patients with classical AIH. Findings were extracted from routine liver pathology reports. The evaluated characteristics included interface hepatitis, lobular hepatitis, portal inflammation, plasma-cell infiltration, eosinophilic infiltration, centrilobular necrosis, confluent necrosis, granulomas, bile-duct injury, cholestasis, steatosis, and fibrosis stage. Histological patterns were classified as AIH-like, predominantly lobular, cholangitic, mixed, or other.

AIH histology was interpreted with reference to the International AIH Pathology Group consensus [25]. Because no single microscopic feature establishes classical AIH, pathology was interpreted with the clinical, biochemical, serological, and imaging data. Features omitted from a routine report were coded as unavailable rather than absent. Histopathological findings were restricted to patients with an available liver biopsy and were summarized descriptively. Because biopsy ascertainment was markedly unequal between groups and slides were not centrally re-reviewed, no formal between-group statistical testing was performed for histopathological variables.

### 2.6. Treatment Response and Outcome Definitions

Early biochemical response was assessed using the documented change in serum aminotransferases within seven days after treatment initiation. A separate early-response endpoint was defined as a ≥50% reduction in aspartate or alanine aminotransferase concentration within 14 days.

Complete biochemical response required normalization of serum aminotransferases and total bilirubin and, when elevated initially and measured subsequently, normalization of IgG. For classical AIH, response was interpreted using IAIHG consensus definitions [22,23,26].

A steroid-refractory course was recorded when persistent or progressive biochemical liver injury or escalation to second-line immunosuppression was documented by the treating team. The source records did not support retrospective application of one uniform corticosteroid dose-duration threshold; this variable was therefore analysed as a clinician-documented treatment characteristic. Relapse was the recorded recurrence of biochemical liver injury after an initial response that led to reintroduction or intensification of immunosuppression after an alternative cause had been excluded.

Recorded clinical outcomes included acute liver failure, hospitalisation, intensive care unit admission, liver transplantation, all-cause death, and liver-related death. Acute liver failure was retained as a clinician-documented endpoint because the study dataset lacked the complete timing and encephalopathy variables required for uniform retrospective re-adjudication. These endpoints were summarized descriptively rather than interpreted as standardized prognostic outcomes.

Follow-up was intended to extend from the index hepatitis diagnosis to the last documented contact, death, transplantation, or end of record. A numeric follow-up duration was available for only 3/40 ICI-H patients and all 39 classical AIH patients; follow-up-dependent endpoints were therefore not compared inferentially.

### 2.7. Derived Inflammatory Indices

The neutrophil-to-lymphocyte ratio was calculated by dividing the absolute neutrophil count by the absolute lymphocyte count.

The C-reactive protein–albumin–lymphocyte index (CALLY index) was calculated from available index-episode laboratory components obtained before corticosteroid or other immunosuppressive treatment [27]:

CALLY index = albumin (g/dL) × absolute lymphocyte count (10^3^/µL)/C-reactive protein (mg/L).

This unit-specific expression is mathematically equivalent to the originally published formulation after conversion of lymphocyte and C-reactive protein units. CALLY was examined only as a continuous exploratory descriptor because it has not been validated for distinguishing ICI-H from classical AIH. No published or data-derived threshold was applied, values were omitted when a component was missing, and no claim of incremental diagnostic value was made.

### 2.8. Statistical Analysis

**Figure 2:**
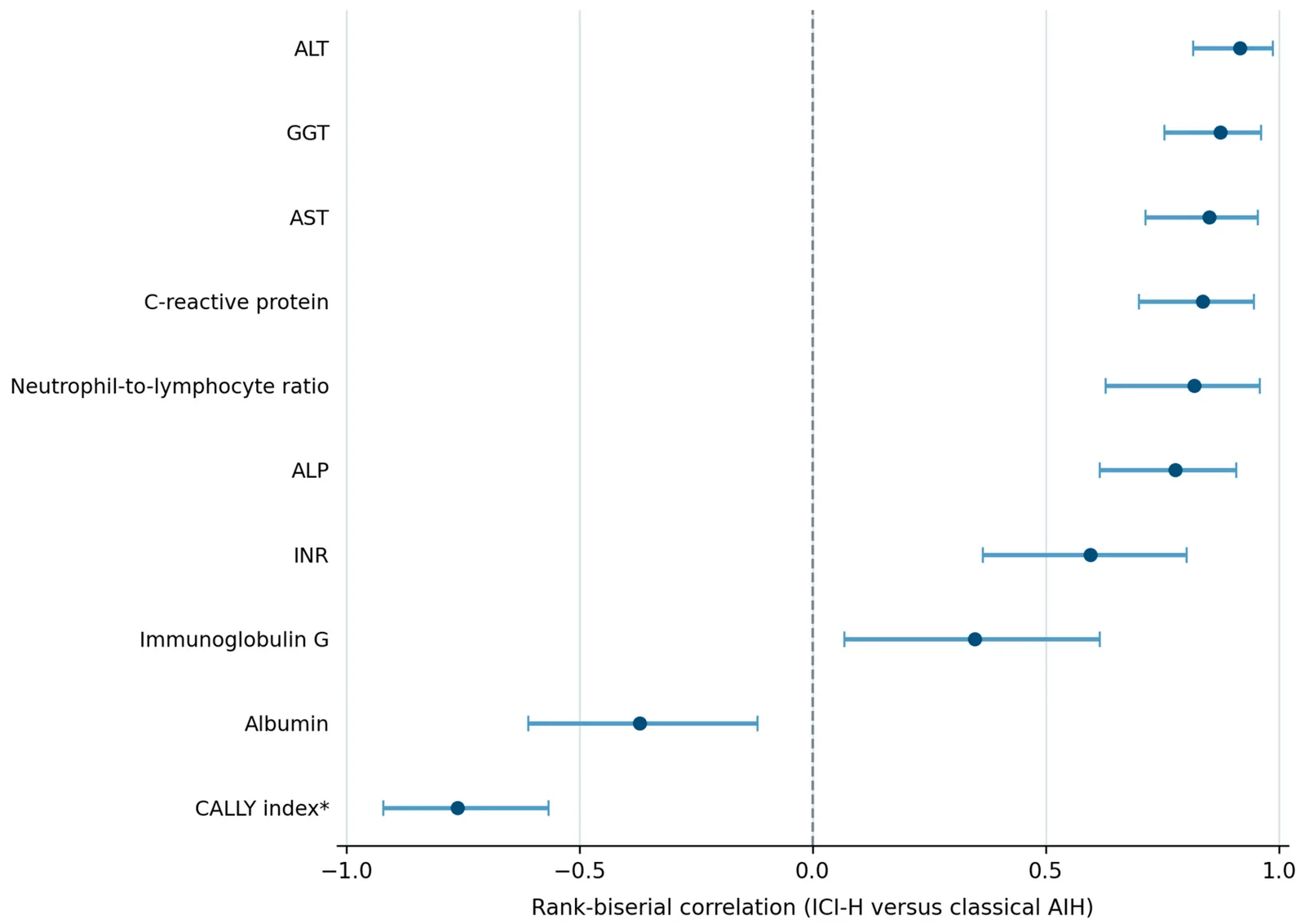
Cross-sectional phenotypic effect sizes. Rank-biserial correlations compare ICI-H with classical AIH; positive values indicate higher measurements in ICI-H and negative values indicate higher measurements in classical AIH. Horizontal lines are 95% percentile bootstrap confidence intervals (20,000 resamples). The asterisk identifies the CALLY index as an exploratory composite descriptor.

Continuous variables were summarized as median [interquartile range] and compared using two-sided Mann–Whitney U tests. Effect estimates are Hodges–Lehmann location shifts (ICI-H minus classical AIH) with approximate 95% confidence intervals derived from the Mann–Whitney distribution with tie correction. Rank-biserial correlations used in Figure 2 were accompanied by 20,000-resample percentile bootstrap confidence intervals.

Categorical variables were reported as numbers and percentages and compared using two-sided Fisher’s exact tests.

The clinical, biochemical, serological, exploratory-index, and treatment families comprised 6, 15, 6, 1, and 4 comparisons, respectively. Histopathological variables were summarized descriptively and were not subjected to between-group hypothesis testing or multiplicity adjustment. Unadjusted *p*-values and adjusted q-values are reported; all tests were two-sided, and q < 0.05 defined statistical significance. Diagnostic scores were descriptive because the simplified AIH score contributed to classical AIH eligibility. Follow-up-dependent and incompletely re-adjudicated clinical outcomes were summarized without hypothesis tests or regression modelling.

No multivariable prognostic model was fitted because follow-up duration was unavailable for most ICI-H patients and key oncological, transplant-eligibility, and endpoint-timing variables could not be harmonized across groups.

No a priori sample-size calculation was undertaken because this was a retrospective review of uncommon conditions; all eligible records in the source population were included. Analyses used complete cases without imputation and were performed in Python 3.12 with SciPy 1.17.0 and reproducible custom code. Monte Carlo and bootstrap procedures used a fixed seed (20260811).

### 2.9. Ethics Approval and Informed Consent

The Bursa Uludağ University Faculty of Medicine Clinical Research Ethics Committee approved this retrospective study on 24 October 2023 (decision no. 2023-21/21). The study was conducted in accordance with the Declaration of Helsinki and applicable institutional requirements.

Written informed consent for the scientific use and publication of de-identified clinical data in this retrospective study was documented for every included patient. The analytical dataset used anonymised study identifiers, and confidentiality was maintained throughout the review and analysis.

## 3. Results

### 3.1. Study Population and Baseline Clinical Characteristics

The cohort included 40 patients with ICI-H and 39 with classical AIH. All index diagnoses occurred between January 2018 and October 2023. Age distributions were similar (48.0 [42.3–55.0] vs. 50.0 [40.0–56.0] years; *p* = 0.926). Men accounted for 26/40 (65.0%) of the ICI-H group and 8/39 (20.5%) of the classical AIH group (OR 7.20, 95% CI 2.61–19.82; *p* < 0.001, q < 0.001).

Diabetes mellitus, hypertension, and acute presentation did not differ significantly between groups. Cirrhosis at index diagnosis was recorded in 0/40 patients with ICI-H and 4/39 (10.3%) with classical AIH; this contrast did not meet the false-discovery rate threshold (*p* = 0.055, q = 0.164). In all four classical AIH patients with cirrhosis, AIH was first formally diagnosed during the index episode; none had a previously established diagnosis of AIH. Acute presentation occurred in 33/40 (82.5%) and 26/39 (66.7%) patients, respectively (*p* = 0.126) (Table 1).

### 3.2. Characteristics of ICI-Related Hepatitis

Accordingly, 39 of 40 patients with ICI-H (97.5%) received a PD-1/PD-L1-pathway inhibitor and one received anti-CTLA-4 therapy. Nivolumab was the most frequent agent (34/40, 85.0%). The median interval from ICI initiation to hepatitis was 15.0 [9.0–36.5] days, and the median exposure was 1.0 [1.0–3.0] treatment cycles (both *n* = 39). Median RUCAM was 7.0 [6.0–8.0]. All 40 ICI-H episodes met CTCAE grade 4 criteria on the basis of ALT elevation (>20 × ULN), with baseline aminotransferase levels within the normal range in all patients.

ICI discontinuation was documented in all 39 patients with available treatment-disposition data. Seventeen of these 39 patients (43.6%) were rechallenged, with recurrent hepatitis in 6/17 (35.3%). Other immune-related adverse events occurred in 14/39 patients (35.9%) (Table 2).

### 3.3. Biochemical and Inflammatory Characteristics

Compared with classical AIH, ICI-H showed higher median AST (1112.5 [843.8–1450.0] vs. 440.0 [242.0–631.5] U/L), ALT (1927.5 [1490.0–2520.0] vs. 426.0 [285.0–713.0] U/L), ALP (457.5 [266.3–595.0] vs. 143.0 [117.5–196.5] U/L), and GGT (980.0 [589.0–1325.0] vs. 99.0 [50.0–211.5] U/L); *p* < 0.001 and q < 0.001 for each comparison. Total and direct bilirubin did not differ significantly.

Systemic inflammatory and coagulation measures were also higher in ICI-H: INR 1.90 [1.60–2.20] versus 1.29 [1.09–1.65], white blood cell count 10.55 [9.25–11.83] versus 6.57 [5.18–7.97] × 10^3^/µL, neutrophils 8.30 [6.88–9.73] versus 3.40 [2.56–4.48] × 10^3^/µL, neutrophil-to-lymphocyte ratio 9.28 [6.74–12.68] versus 2.25 [1.73–3.33], and C-reactive protein 75.0 [44.3–105.8] versus 6.1 [2.4–17.8] mg/L (all *p* < 0.001, q < 0.001).

Albumin (2.9 [2.6–3.1] vs. 3.2 [2.9–4.7] g/dL; *p* = 0.005, q = 0.006) and absolute lymphocytes (0.90 [0.70–1.00] vs. 1.42 [0.76–1.78] × 10^3^/µL; *p* = 0.003, q = 0.004) were lower in ICI-H. Platelets were higher in ICI-H (177.5 [149.5–211.3] vs. 104.0 [69.1–245.8] × 10^3^/µL; *p* = 0.036, q = 0.042).

IgG measurements were available for all 40 patients with ICI-H and 32 patients with classical AIH. In this available-case comparison, median IgG was 2455.0 [2150.0–2762.5] and 2005.0 [1507.5–2475.0] mg/dL, respectively (*p* = 0.012, q = 0.015).

### 3.4. Serological Findings and Diagnostic Descriptors

ANA was positive in 5/40 (12.5%) patients with ICI-H and 29/39 (74.4%) with classical AIH (OR 0.05, 95% CI 0.02–0.16; *p* < 0.001, q < 0.001). Positivity for ASMA, AMA, anti-LKM, anti-SLA/LP, and pANCA did not differ significantly. Tests recorded as not performed were excluded from their respective denominators.

AIH-related diagnostic scores were summarized descriptively in the biopsy-assessed subgroup only. The simplified score was 5.5 [4.3–6.8] in 10 ICI-H biopsies and 7.0 [7.0–7.0] in 39 classical AIH biopsies; the corresponding revised IAIHG scores were 14.0 [11.3–15.0] and 15.0 [14.0–16.0]. No inferential comparison was performed because the simplified score contributed to classical AIH eligibility, and biopsy availability differed between groups.

CALLY was calculable in 76 patients (40 ICI-H; 36 classical AIH). The median index was lower in ICI-H (0.0353 [0.0195–0.0743] vs. 0.8140 [0.2317–1.7940]; *p* < 0.001, q < 0.001; rank-biserial r = −0.761, 95% CI −0.921 to −0.567). Because each component already differed between groups, CALLY was interpreted as a composite phenotype descriptor rather than an independent biomarker (Table 3).

### 3.5. Histopathological Findings

Biopsy data were available for 10/40 (25.0%) ICI-H patients and all 39 classical AIH patients. Because biopsy ascertainment was markedly unequal between groups, histopathological findings are presented descriptively without formal between-group statistical testing. Median interface-hepatitis grade was 0.0 [0.0–0.8] in ICI-H and 2.0 [1.5–3.0] in classical AIH, while portal-inflammation grades were 0.0 [0.0–1.0] and 2.0 [1.0–2.0], respectively. Eosinophilic infiltration was present in 3/10 (30.0%) ICI-H biopsies and in none of the 39 classical AIH biopsies. Other histopathological features and overall pattern distributions are summarized descriptively in Table 4.

### 3.6. Treatment and Recorded Outcomes

All 79 patients received corticosteroids. A clinician-documented steroid-refractory course occurred in 14/40 (35.0%) ICI-H and 14/39 (35.9%) classical AIH patients. Second-line immunosuppression was used in 11/40 (27.5%) and 14/39 (35.9%), respectively. None of the four prespecified treatment comparisons met the false-discovery-rate threshold (Table 5).

Clinically recorded outcomes are presented as descriptive cumulative proportions. Relapse was documented in 14/40 (35.0%) ICI-H and 25/39 (64.1%) classical AIH patients. Clinician-documented acute liver failure occurred in 11/40 (27.5%) and 24/39 (61.5%), and liver transplantation in 0/40 and 9/39 (23.1%), respectively. These contrasts were not tested inferentially because follow-up, endpoint re-adjudication, referral patterns, and transplant eligibility were not comparable.

Hospitalisation was recorded in 22/40 (55.0%) ICI-H and 24/39 (61.5%) classical AIH patients; intensive care unit admission in 14/40 (35.0%) and 22/39 (56.4%); all-cause death in 12/40 (30.0%) and 11/39 (28.2%); and liver-related death in 4/40 (10.0%) and 4/39 (10.3%), respectively. These unstandardized recorded outcomes were not interpreted as comparative risks.

No adjusted outcome models were retained because the available dataset could not support comparable time at risk or adequate adjustment for malignancy, referral, liver reserve, and transplant eligibility.

Follow-up duration was available for only 3/40 ICI-H patients, compared with all 39 classical AIH patients. The recorded outcome proportions in Table 5 are therefore descriptive and should not be interpreted as incidence, hazard, or comparative prognosis. Figure 2 instead summarizes robust cross-sectional phenotype effect sizes.

### 3.7. Completeness and Interpretation of Longitudinal Outcomes

Early biochemical-response variables were nearly complete in ICI-H but were documented for only one to ten classical AIH patients; no between-group response analysis was performed. Follow-up duration was available for 3/40 ICI-H and 39/39 classical AIH patients.

Overall survival, progression-free survival, and time-to-relapse variables were unavailable. Kaplan–Meier, log-rank, Cox, and follow-up-independent logistic outcome analyses were therefore not undertaken.

## 4. Discussion

In this comparison, ICI-H and classical AIH showed different observed clinical, biochemical, and serological profiles, with additional descriptive histopathological patterns in the biopsy-assessed subgroup. These between-group differences should be interpreted in the context of the disease-specific criteria used for cohort assignment and therefore do not represent independent diagnostic discriminators. ICI-H occurred predominantly in men and was associated with greater liver-enzyme elevation and systemic inflammation, lower albumin and lymphocyte concentrations, and less frequent ANA positivity. Histopathological observations were limited to a selectively biopsied subgroup and were not used to support between-group inference. Taken together, these phenotype-level findings support consideration of ICI-H as a heterogeneous immune-mediated drug-induced liver injury rather than an ICI-triggered form of classical AIH. Treatment and recorded outcome data were descriptive and did not establish comparative prognosis.

The present findings should be interpreted as an extension and regional validation of previous comparative studies rather than as evidence of an entirely novel phenotype. Earlier reports have demonstrated differences between ICI-associated liver injury and classical AIH, particularly in histopathological and serological characteristics [10,11,12]. However, these studies have generally been histology-enriched, have included additional comparator groups, or have evaluated relatively small ICI-associated cohorts. In contrast, the present study provides a direct comparison of 40 patients with ICI-H and 39 with classical AIH across several complementary clinical and laboratory domains, without restricting the overall ICI-H cohort to patients who underwent liver biopsy. The inclusion of a cohort from Türkiye also increases the geographic diversity of the available evidence. Thus, the main contribution of this study is the multidomain characterization and contextual validation of previously reported distinctions between ICI-H and classical AIH in a real-world regional cohort.

The biochemical contrast was substantial: median AST, ALT, ALP, and GGT were all higher in ICI-H, whereas bilirubin did not differ. However, the magnitude of these biochemical contrasts should be interpreted cautiously because eligibility for the ICI-H cohort required an updated RUCAM score of ≥6. Although RUCAM is a multidomain causality-assessment tool rather than a direct severity scale, its use in cohort assignment may have preferentially selected episodes with a more coherent drug-related biochemical pattern and thereby contributed to the observed enzyme-profile differences. Previous cohorts likewise describe hepatocellular, mixed, and cholestatic phenotypes and show that marked aminotransferase elevation may occur without parallel hyperbilirubinaemia or irreversible hepatic dysfunction [1,2,3,4,5]. Accordingly, AST or ALT alone should not be used to infer mechanism or longer-term prognosis.

ICI-H also occurred in a more inflammatory clinical context, with higher CRP, neutrophil count, neutrophil-to-lymphocyte ratio, and INR and lower albumin and lymphocyte concentrations. These findings cannot be attributed solely to hepatic immune injury. Active cancer, intercurrent infection or inflammation, nutritional status, previous oncological treatment, and corticosteroid exposure may all contribute. The male predominance should be interpreted with the same caution because the sex distribution of the underlying cancers and ICI indications was not balanced between groups.

Serological profiles also differed descriptively between groups. ANA positivity was much less frequent in ICI-H, in keeping with reports that conventional autoantibodies have limited sensitivity and uncertain specificity in checkpoint inhibitor-induced liver injury [2,9,12]. A previous cancer cohort also found that baseline autoantibody positivity did not reliably predict later ICI-associated hepatitis [9]. Negative ANA or ASMA therefore does not exclude ICI-H, and isolated seropositivity after ICI exposure does not establish classical AIH.

The higher median absolute IgG concentration in the available ICI-H cases was less consistent with published series, in which IgG is often normal or only moderately elevated [2,12]. Seven classical AIH patients lacked an IgG measurement; values were analysed as absolute concentrations rather than uniform multiples of the laboratory upper limit of normal; and sampling time may have differed. This available-case result may therefore reflect selection and measurement differences and should not be read as evidence of a stronger autoimmune phenotype in ICI-H.

AIH diagnostic scores require particularly careful interpretation. The simplified score contributed to classical AIH eligibility, while biopsy information was available for only one-quarter of ICI-H cases. We therefore restricted score summaries to the biopsy-assessed subgroup and did not perform an inferential comparison. The scores were developed to classify classical AIH after competing diagnoses are excluded, not to diagnose checkpoint inhibitor-induced injury [6,7,24]. A high score after ICI exposure does not by itself establish classical AIH, and a low score does not exclude ICI-H [18,22,23].

Histopathological findings were considered exploratory observations limited to the biopsy-assessed subgroup. Because biopsy ascertainment was markedly asymmetric and non-random, the observed differences in interface inflammation, portal inflammation, and eosinophilic infiltration cannot support reliable between-group histological inference. These descriptive observations are therefore hypothesis-generating only. Previously reported histological patterns of ICI-associated liver injury include lobular or centrilobular inflammation, granulomas, endothelial injury, bile-duct damage, and CD8-positive T-cell predominance [10,11,12,13,14,15], but the present dataset was not suitable for confirming such distinctions.

The biopsy results are vulnerable to selection bias. Only 10 of 40 ICI-H patients underwent biopsy, compared with all 39 classical AIH patients, so the ICI-H specimens may overrepresent atypical, severe, cholestatic, or treatment-refractory cases. Histological features were abstracted from routine reports without blinded central re-review, and biopsy timing relative to corticosteroid exposure was not standardised [14]. Apparent similarities in other microscopic features should not be interpreted as evidence of equivalence, particularly with such unequal biopsy ascertainment. Pathology consensus also stresses that no single microscopic feature is specific enough to diagnose AIH outside its clinical and serological context [25].

Every included patient received corticosteroids, and about one-third of each group had a steroid-refractory course. More than one-quarter of ICI-H patients required second-line treatment. Mycophenolate mofetil is the most frequently described option for steroid-refractory ICI-H, although the evidence comes largely from retrospective series rather than randomised comparisons [5,13,16]. Because there was no untreated ICI-H comparator, these data cannot identify patients who might have improved after ICI withdrawal alone or determine the effect of steroid dose, timing, and duration.

Recorded relapse, acute liver failure, and transplantation differed numerically between groups, but these contrasts cannot be interpreted as comparative risks. Follow-up duration was missing for 37/40 ICI-H patients, time-to-event variables were unavailable, acute liver failure could not be uniformly re-adjudicated, and active malignancy may preclude transplantation. The outcome data therefore describe what was present in the available records rather than the incidence or natural history of either disease.

Hepatitis recurred in 6 of the 17 rechallenged patients. Re-exposure thus carried a clinically relevant but non-universal risk, consistent with the variable course reported in multicentre cohorts [3,4]. Rechallenge should be individualised according to the severity and phenotype of the initial episode, response to immunosuppression, oncological alternatives, and the expected benefit of renewed ICI therapy, with coordinated oncology and hepatology follow-up.

The recorded outcome profile must also be considered in the context of referral and competing risks. Classical AIH patients were drawn from a tertiary-care population that may overrepresent acute severe or advanced disease, whereas cancer progression, infection, treatment exposure, and non-hepatic causes may dominate outcomes in ICI-H. Direct prognostic comparisons require standardized entry points, endpoint adjudication, transplant-eligibility data, and complete longitudinal follow-up.

CALLY was markedly lower in ICI-H but remains an exploratory result. The index was developed as an oncological prognostic measure combining CRP, albumin, and lymphocyte count [27]. All three components already differed between the present groups, so the composite largely restates those component-level contrasts. It should not be considered an independent diagnostic biomarker, and no validated cutoff exists for separating ICI-H from classical AIH. Pre-ICI CALLY values were not available in the study dataset, precluding assessment of within-patient change from the pre-ICI state to the index hepatitis episode. Therefore, the observed CALLY and NLR differences may reflect the underlying malignancy and oncological treatment, as well as systemic inflammation accompanying ICI-H, and should not be interpreted as specific markers of the hepatitis phenotype. External evaluation and evidence of added value beyond routine biochemical and serological measures are needed before clinical use.

The study benefits from comparison of both disorders within one centre, a common data dictionary, standardized units and missing-data rules, updated RUCAM-based causality assessment for ICI-H, and established diagnostic criteria for classical AIH. Effect estimates and false-discovery-rate correction for the prespecified non-histopathological comparison families reduced reliance on unadjusted *p*-values.

The main limitations are the retrospective single-centre design, modest sample size, selection from records already labelled as autoimmune or immune-mediated hepatitis, and retrospective assignment of mutually exclusive primary exclusion reasons for the 21 non-included records. The multiyear study period may have introduced temporal heterogeneity. Biopsy ascertainment was markedly unequal, pathology was not centrally re-reviewed, and diagnostic-score interpretation was constrained by incorporation and missing-component bias. Although updated RUCAM provided a structured and predefined framework for causality assessment, it was not specifically developed or validated for immune-mediated liver injury caused by ICIs. Accordingly, requiring a RUCAM score of ≥6 may have excluded some clinically plausible cases of ICI-related liver injury; the four ICI-exposed cases below this threshold were therefore reviewed individually and are described in Supplementary Table S1. In addition, use of this threshold may have influenced the observed enzyme-profile differences by preferentially retaining cases with a more coherent drug-related biochemical pattern; therefore, the magnitude of these contrasts should not be interpreted as a purely biological separation between ICI-H and classical AIH. Pretreatment sampling windows were not uniform. Follow-up was incomplete in ICI-H, early response data were sparse in classical AIH, and several outcomes could not be uniformly re-adjudicated. Active cancer, treatment exposure, referral patterns, and transplant eligibility produced residual confounding that precluded causal or prognostic inference. Independent cohorts with uniformly harmonized data collection are required for validation.

The practical implication is that ICI-H and classical AIH should not be used as interchangeable labels. After ICI exposure, severe liver-enzyme elevation with negative conventional serology can still represent immune-mediated injury. Conversely, autoantibody positivity or an elevated IgG concentration does not by itself prove classical AIH. Diagnosis should integrate the exposure timeline, exclusion of competing causes, biochemical phenotype, imaging, causality assessment, and clinical course [5,18,19,20]. Biopsy is most useful when the presentation is atypical, cholestatic, severe, diagnostically uncertain, or insufficiently responsive to treatment [5,10,14,25]. Classical AIH requires separate recognition of its chronic relapsing course and potential need for long-term maintenance immunosuppression [6,22,23].

## 5. Conclusions

In this single-centre cohort, ICI-H and classical AIH showed different observed clinical, biochemical, and serological profiles, with descriptive histopathological patterns in the biopsy-assessed subgroup. ICI-H showed greater liver-enzyme elevation and systemic inflammation, whereas classical AIH showed more frequent ANA positivity. Histopathological observations were limited by markedly unequal and non-random biopsy ascertainment and should therefore be regarded as exploratory only. CALLY summarized differences already present in CRP, albumin, and lymphocyte components and remains exploratory. Because longitudinal follow-up and outcome ascertainment were not comparable, this study does not establish prognostic differences. Because disease-specific diagnostic criteria contributed to cohort assignment, the observed between-group differences should not be interpreted as independent diagnostic discriminators. ICI-H should be evaluated as a distinct immune-mediated liver injury using exposure timing, exclusion of competing causes, structured causality assessment, serology, and selective histology rather than classical AIH criteria alone.

## Figures and Tables

**Table 1:** Baseline clinical characteristics of the study groups.

Variable	ICI-H (*n* = 40)	Classical AIH (*n* = 39)	Effect Estimate (95% CI)	*p*/q
Age, years	48.0 [42.2–55.0]	50.0 [40.0–56.0]	HL 0.00 (−6.00–5.00)	*p* = 0.926 q = 1.000
Male sex	26/40 (65.0%)	8/39 (20.5%)	OR 7.20 (2.61–19.82)	*p* < 0.001 q < 0.001
Diabetes mellitus	4/40 (10.0%)	7/39 (17.9%)	OR 0.51 (0.14–1.90)	*p* = 0.348 q = 0.522
Hypertension	4/40 (10.0%)	4/39 (10.3%)	OR 0.97 (0.23–4.19)	*p* = 1.000 q = 1.000
Cirrhosis at index diagnosis	0/40 (0.0%)	4/39 (10.3%)	OR 0.10 (0.01–1.87)	*p* = 0.055 q = 0.164
Acute presentation	33/40 (82.5%)	26/39 (66.7%)	OR 2.36 (0.82–6.76)	*p* = 0.126 q = 0.253

Data are presented as median [interquartile range] or *n*/*N* (%). HL is the Hodges–Lehmann location shift (ICI-H minus classical AIH); OR is the odds ratio for ICI-H versus classical AIH. Continuous variables were analyzed using two-sided Mann–Whitney U tests, and binary variables were analyzed using two-sided Fisher’s exact tests. The q-values are Benjamini–Hochberg-adjusted across the six clinical comparisons shown.

**Table 2:** Oncology and treatment characteristics of the ICI-H group.

Characteristic	ICI-H (*n* = 40)
Primary malignancy, *n*/*N* (%)	Lung 16/39 (41.0%); melanoma 13/39 (33.3%); renal cell carcinoma 6/39 (15.4%); gastrointestinal 3/39 (7.7%); other 1/39 (2.6%)
Cancer stage, *n*/*N* (%)	Localized 20/40 (50.0%); locally advanced 14/40 (35.0%); metastatic 6/40 (15.0%)
Liver metastasis	4/40 (10.0%)
ICI pathway/class	PD-1/PD-L1 pathway 39/40 (97.5%); CTLA-4 1/40 (2.5%)
ICI agent	Nivolumab 34/40 (85.0%); pembrolizumab 4/40 (10.0%); atezolizumab 1/40 (2.5%); ipilimumab 1/40 (2.5%)
Interval from ICI initiation to hepatitis, days	15.0 [9.0–36.5] (*n* = 39)
Cycles before hepatitis	1.0 [1.0–3.0] (*n* = 39)
Updated RUCAM score	7.0 [6.0–8.0]
CTCAE grade	Grade 4: 40/40 (100.0%)
Concurrent immune-related adverse event	14/39 (35.9%)
ICI discontinued	39/39 (100%)
ICI rechallenge	17/39 (43.6%)
Recurrent hepatitis after rechallenge	6/17 (35.3%)

Data are presented as median [interquartile range] or *n*/*N* (%). Denominators are shown when data were missing. As this table describes the ICI-H group only, comparative effect estimates and *p*/q-values are not applicable. ICI, immune checkpoint inhibitor; CTCAE, Common Terminology Criteria for Adverse Events; RUCAM, Roussel Uclaf Causality Assessment Method.

**Table 3:** Biochemical, serological, and diagnostic-descriptor comparison.

Variable	ICI-H (*n* = 40)	Classical AIH (*n* = 39)	Effect Estimate (95% CI)	*p*/q
AST, U/L	1112.5 [843.8–1450.0]	440.0 [242.0–631.5]	HL 673.50 (536.00–853.00)	***p* < 0.001** **q < 0.001**
ALT, U/L	1927.5 [1490.0–2520.0]	426.0 [285.0–713.0]	HL 1406.00 (1182.00–1712.00)	***p* < 0.001** **q < 0.001**
ALP, U/L	457.5 [266.2–595.0]	143.0 [117.5–196.5]	HL 298.00 (207.00–364.00)	***p* < 0.001** **q < 0.001**
GGT, U/L	980.0 [589.0–1325.0]	99.0 [50.0–211.5]	HL 804.00 (609.00–977.00)	***p* < 0.001** **q < 0.001**
Total bilirubin, mg/dL	7.2 [4.8–10.4]	5.7 [1.6–13.4]	HL 2.00 (−1.30–3.90)	*p* = 0.178 q = 0.178
Direct bilirubin, mg/dL	4.9 [3.1–7.2]	3.1 [0.7–10.6]	HL 1.51 (−0.90–2.90)	*p* = 0.164 q = 0.175
INR	1.90 [1.60–2.20]	1.29 [1.09–1.65] *(n* = 36)	HL 0.56 (0.37–0.72)	***p* < 0.001** **q < 0.001**
Albumin, g/dL	2.9 [2.6–3.1]	3.2 [2.9–4.7]	HL −0.50 (−1.20–−0.20)	***p* = 0.005** **q = 0.006**
Platelet count, ×10^3^/µL	177.5 [149.5–211.2]	104.0 [69.1–245.8]	HL 54.30 (5.00–87.10)	***p* = 0.036** **q = 0.042**
White blood cell count, ×10^3^/µL	10.6 [9.2–11.8]	6.6 [5.2–8.0]	HL 3.96 (2.96–4.93)	***p* < 0.001** **q < 0.001**
Neutrophil count, ×10^3^/µL	8.3 [6.9–9.7]	3.4 [2.6–4.5] (*n* = 28)	HL 4.78 (3.94–5.77)	***p* < 0.001** **q < 0.001**
Lymphocyte count, ×10^3^/µL	0.9 [0.7–1.0]	1.4 [0.8–1.8] (*n* = 36)	HL −0.52 (−0.77–−0.18)	***p* = 0.003** **q = 0.004**
Neutrophil-to-lymphocyte ratio	9.28 [6.74–12.68]	2.25 [1.73–3.33] (*n* = 28)	HL 6.35 (4.70–8.74)	***p* < 0.001** **q < 0.001**
C-reactive protein, mg/L	75.0 [44.2–105.8]	6.1 [2.3–17.8]	HL 59.50 (40.60–80.60)	***p* < 0.001** **q < 0.001**
Immunoglobulin G, mg/dL	2455.0 [2150.0–2762.5]	2005.0 [1507.5–2475.0] (*n* = 32)	HL 435.00 (120.00–760.00)	***p* = 0.012** **q = 0.015**
ANA positivity	5/40 (12.5%)	29/39 (74.4%)	OR 0.05 (0.02–0.16)	***p* < 0.001** **q < 0.001**
ASMA positivity	3/40 (7.5%)	2/39 (5.1%)	OR 1.50 (0.24–9.50)	*p* = 1.000 q = 1.000
AMA positivity	0/40 (0.0%)	2/39 (5.1%)	OR 0.19 (0.01–3.98)	*p* = 0.241 q = 0.481
Anti-LKM positivity	1/40 (2.5%)	4/39 (10.3%)	OR 0.22 (0.02–2.10)	*p* = 0.201 q = 0.481
Anti-SLA/LP positivity	1/40 (2.5%)	3/39 (7.7%)	OR 0.31 (0.03–3.09)	*p* = 0.359 q = 0.538
pANCA positivity	1/40 (2.5%)	1/39 (2.6%)	OR 0.97 (0.06–16.15)	*p* = 1.000 q = 1.000
Simplified AIH score, biopsied subgroup, points	5.5 [4.3–6.8] (*n* = 10)	7.0 [7.0–7.0] (*n* = 39)	Descriptive only	—
Revised IAIHG score, biopsied subgroup, points	14.0 [11.3–15.0] (*n* = 10)	15.0 [14.0–16.0] (*n* = 39)	Descriptive only	—
CALLY index	0.0353 [0.0195–0.0743]	0.8140 [0.2317–1.7940] (*n* = 36)	r_rb −0.761 (−0.921–−0.567)	***p* < 0.001** **q < 0.001**

Data are presented as median [interquartile range] or *n*/*N* (%). Denominators below the group total are shown where data were missing. HL is the Hodges–Lehmann location shift; OR is the odds ratio; r_rb is the rank-biserial correlation. Diagnostic scores are descriptive in the biopsy-assessed subgroup and were not tested. CALLY was analysed as an exploratory continuous descriptor without a cutoff. The q-values are Benjamini–Hochberg-adjusted within the relevant biochemical, serological, or exploratory-index family. Bold *p*/q values indicate comparisons meeting the statistical significance threshold after Benjamini–Hochberg adjustment (q < 0.05).

**Table 4:** Histopathological findings in patients with an available liver biopsy.

Variable	ICI-H Biopsies (*n* = 10)	Classical AIH Biopsies (*n* = 39)
Liver biopsy available	10/40 (25.0%)	39/39 (100.0%)
Interface hepatitis grade	0.0 [0.0–0.8] (*n* = 10)	2.0 [1.5–3.0]
Lobular hepatitis grade	2.0 [1.0–2.0] (*n* = 10)	1.0 [0.0–2.0]
Portal inflammation grade	0.0 [0.0–1.0] (*n* = 10)	2.0 [1.0–2.0]
Plasma-cell infiltration grade	1.5 [1.0–2.0] (*n* = 10)	1.0 [0.0–2.0]
Eosinophilic infiltration present	3/10 (30.0%)	0/39 (0.0%)
Steatosis grade	0.0 [0.0–1.0] (*n* = 10)	0.0 [0.0–0.0]
Fibrosis stage	2.0 [1.0–2.8] (*n* = 10)	2.0 [2.0–3.0]
Centrilobular necrosis	4/10 (40.0%)	12/39 (30.8%)
Confluent necrosis	5/10 (50.0%)	14/39 (35.9%)
Granuloma	0/10 (0.0%)	0/39 (0.0%)
Bile-duct injury	1/10 (10.0%)	9/39 (23.1%)
Cholestasis	5/10 (50.0%)	12/39 (30.8%)
Histologic pattern	AIH-like 3; Lobular 2; Cholangitic 3; Mixed 2	AIH-like 17; Lobular 8; Cholangitic 8; Mixed 6

Data are presented descriptively as median [interquartile range], *n*/*N* (%), or counts, as appropriate. No formal between-group statistical testing was performed for histopathological variables because biopsy ascertainment was markedly unequal between groups.

**Table 5:** Treatment characteristics and recorded clinical outcomes.

Variable	ICI-H (*n* = 40)	Classical AIH (*n* = 39)	Effect Estimate (95% CI)	*p*/q
Corticosteroid use	40/40 (100.0%)	39/39 (100.0%)	Not estimable †	No test †
Mycophenolate mofetil use	5/40 (12.5%)	11/39 (28.2%)	OR 0.36 (0.11–1.17)	*p* = 0.099 q = 0.396
Ursodeoxycholic acid use	22/40 (55.0%)	18/39 (46.2%)	OR 1.43 (0.59–3.46)	*p* = 0.503 q = 0.671
Steroid-refractory course	14/40 (35.0%)	14/39 (35.9%)	OR 0.96 (0.38–2.42)	*p* = 1.000 q = 1.000
Second-line immunosuppression	11/40 (27.5%)	14/39 (35.9%)	OR 0.68 (0.26–1.76)	*p* = 0.474 q = 0.671
Relapse	14/40 (35.0%)	25/39 (64.1%)	Descriptive ‡	No test ‡
Acute liver failure	11/40 (27.5%)	24/39 (61.5%)	Descriptive ‡	No test ‡
Hospitalisation	22/40 (55.0%)	24/39 (61.5%)	Descriptive ‡	No test ‡
Intensive care unit admission	14/40 (35.0%)	22/39 (56.4%)	Descriptive ‡	No test ‡
Liver transplantation	0/40 (0.0%)	9/39 (23.1%)	Descriptive ‡	No test ‡
All-cause death	12/40 (30.0%)	11/39 (28.2%)	Descriptive ‡	No test ‡
Liver-related death	4/40 (10.0%)	4/39 (10.3%)	Descriptive ‡	No test ‡

Data are *n*/*N* (%). † The odds ratio is not estimable, and no test was performed because corticosteroid use was 100% in both groups. Four other treatment characteristics used two-sided Fisher’s exact tests with Benjamini–Hochberg adjustment. ‡ Recorded outcomes are descriptive only; effect estimates and tests were omitted because follow-up and endpoint ascertainment were not comparable.

## Data Availability

The datasets generated and analyzed during the current study are available from the corresponding author upon reasonable request, subject to institutional and ethics approval. The data are not publicly available because they contain potentially sensitive clinical information.

## Supplementary Materials

The following supporting information can be downloaded at https://www.mdpi.com/article/10.3390/jcm15197572/s1, Table S1: Clinical and biochemical characteristics of the four ICI-exposed patients excluded because of an updated RUCAM score of <6.
