# Supplementary material for: Immune Checkpoint Inhibitor-Related Hepatitis Versus Classical Autoimmune Hepatitis: A Retrospective Multidomain Phenotype Comparison
Source: J Clin Med. 2026 Sep 29;15(19):7572. doi: 10.3390/jcm15197572 (PMC13666208; doi:10.3390/jcm15197572)
Supplement: Supplementary file 1 [file jcm-15-07572-s001.zip › jcm-4531960-supplementary.pdf]

Supplementary Table S1

Clinical and biochemical characteristics of the four ICI-exposed patients excluded because of an updated RUCAM score <6

Case	ICI agent	ICI initiation date	Liver-injury date	Interval from ICI initiation to liver injury, days	ALT (U/L)	ALP (U/L)	Updated RUCAM
A	Nivolumab	01 Feb 2022	15 Mar 2022	42	356	145	<6
B	Nivolumab	15 Jun 2021	08 Jul 2021	23	482	210	<6
C	Nivolumab	10 Jan 2020	22 Nov 2020	317	275	132	<6
D	Nivolumab	12 Aug 2022	04 Feb 2023	176	615	180	<6

Abbreviations: ALT, alanine aminotransferase; ALP, alkaline phosphatase; ICI, immune checkpoint inhibitor; RUCAM, Roussel Uclaf Causality Assessment Method.

All four patients were excluded from the primary analytical ICI-H cohort because they did not meet the prespecified updated RUCAM threshold of ≥6. The exact component-level RUCAM scores were not retained in the final de-identified analytical dataset.
